# Automated alignment method for coherence-controlled holographic microscope

**DOI:** 10.1117/1.JBO.20.11.111215

**Published:** 2015-10-28

**Authors:** Zbynek Dostal, Tomas Slaby, Lukas Kvasnica, Martin Lostak, Aneta Krizova, Radim Chmelik

**Affiliations:** a Brno University of Technology, Institute of Physical Engineering, Faculty of Mechanical Engineering, Technicka 2896/2, Brno 61600, Czech Republic; b Brno University of Technology, CEITEC-Central European Institute of Technology, Technicka 3058/10, Brno 61600, Czech Republic; c TESCAN ORSAY HOLDING a.s., Libusina trida 21, Brno 623 00, Czech Republic

**Keywords:** holographic microscopy, quantitative phase imaging, automated alignment, holographic signal

## Abstract

A coherence-controlled holographic microscope (CCHM) was developed particularly for quantitative phase imaging and measurement of live cell dynamics, which is the proper subject of digital holographic microscopy (DHM). CCHM in low-coherence mode extends DHM in the study of living cells. However, this advantage is compensated by sensitivity of the system to easily become misaligned, which is a serious hindrance to wanted performance. Therefore, it became clear that introduction of a self-correcting system is inevitable. Accordingly, we had to devise a theory of a suitable control and design an automated alignment system for CCHM. The modulus of the reconstructed holographic signal was identified as a significant variable for guiding the alignment procedures. From this, we derived the original basic realignment three-dimensional algorithm, which encompasses a unique set of procedures for automated alignment that contains processes for initial and advanced alignment as well as long-term maintenance of microscope tuning. All of these procedures were applied to a functioning microscope and the tested processes were successfully validated. Finally, in such a way, CCHM is enabled to substantially contribute to study of biology, particularly of cancer cells *in vitro*.

## 1 Introduction

The coherence-controlled holographic microscope (CCHM)^1^^,^^2^ is an innovative system particularly designed for quantitative phase imaging (QPI) and measurement of live cell dynamics.^3^ An achromatic off-axis interferometer based on the diffraction grating is used in CCHM. The spatial and temporal coherence of illumination can be widely varied, and in this way, imaging properties of the microscope are substantially modifiable. The coherence gating effect induced by low coherence makes possible formation of optical sections of the sample^4^ (in reflection mode) or imaging through turbid media^5^^,^^6^ (in transmission mode). Low coherence also improves lateral resolution and the imaging in general.^7^ For exploiting these effects, CCHM works with a broad polychromatic light source, which makes all the difference to other off-axis holographic microscopes usually equipped with a laser light source.^8^^,^^9^ This is because the high-coherence light source leads to the formation of unwanted artifacts in QPI as a consequence of coherence noise, random interferences, and diffraction of light. Low-coherence illumination, however, requires precise alignment and high stability of the system to be maintained during long-lasting time-lapse QPI studies of activity and reaction of living cells. And just these advantages stand for the contribution of CCHM to cell biology research. For alignment of highly sensitive interferometric systems, a secondary light source is often used. The interferometer state detection is carried out by an auxiliary detector^10^ or by a system of detectors.^11^^–^^13^ These methods are unsuitable for CCHM, because they would cause photo-toxic effect on living cells,^10^^,^^11^^,^^13^ and because the alignment of the many additional optical elements would be very complicated.^12^ For this reason, an original method based on the measurement of the modulus $\overset{¯}{w}$ of the reconstructed holographic signal was elaborated for assessing the instrument state and guiding the optimization. The basic realignment three-dimensional algorithm (BReTA) method is suitable for CCHM because it does not require additional optical components and light sources. Moreover, it is amenable to full automation because the value of the reconstructed holographic signal is available from the online image processing and the operation can be robotized. Finally, the verification of the BReTA method applicability is presented.

## 2 Methods and Materials

### 2.1 Optical Setup and Image Processing

**Fig. 1:**
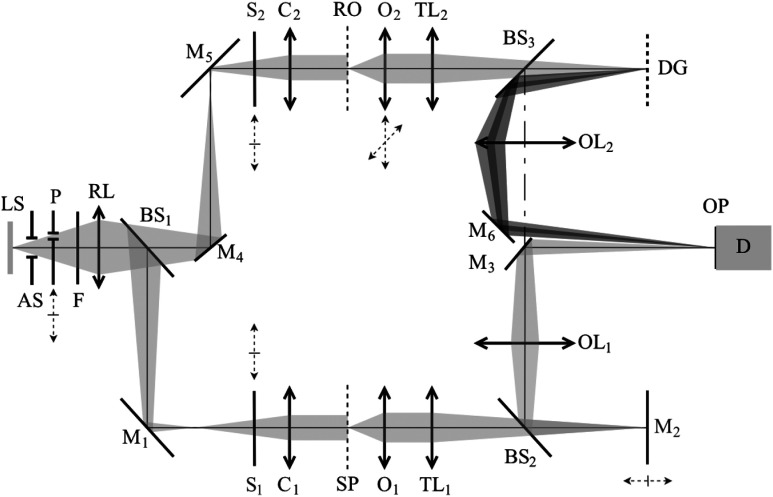
Optical setup of the coherence-controlled holographic microscope (CCHM). LS, light source; AS, aperture stop; F, filter; RL, relay lens; M, mirrors; C, condensers; SP, specimen; RO, reference object; O, objectives; DG, diffraction grating; BS, beam splitters; OL, output lenses; OP, output plane; D, detector (CCD camera). The motorized parts of CCHM: mechanism for inserting a sighting pattern P, computer-controlled shutters S, two-dimensional transversal linear stage of objective $O_{2}$, and linear stage with the axial movement direction of the mirror $M_{2}$.

The optical assembly of CCHM is shown in Fig. 1. A halogen lamp is used as a light source; its light is guided by an optical fiber to the plane light source. This plane is imaged by the relay lens RL into the rear focal plane of condensers $C_{1}$ and $C_{2}$ so that the sample SP and the reference object RO are Köhler illuminated. The light beam is split by beam splitter ${\mathrm{BS}}_{1}$ and mirrors $M_{1}$, $M_{4}$, and $M_{5}$ to the optical paths of object and reference arm. Changeable aperture stop AS is placed into the beam for setting spatial coherence of the light and bandpass filter F for setting its temporal coherence. The specimen and the reference object are imaged from the object plane to the output plane OP by the objective lens $O_{1}$ and $O_{2}$, tube lens ${\mathrm{TL}}_{1}$ and ${\mathrm{TL}}_{2}$, and output lens ${\mathrm{OL}}_{1}$ and ${\mathrm{OL}}_{2}$, respectively. The hologram is captured by the CCD camera D in the output plane OP. Diffraction grating DG and mirror $M_{2}$ are placed in the intermediate image planes behind the tube lenses ${\mathrm{TL}}_{1}$ and ${\mathrm{TL}}_{2}$ to form an interference pattern with the same spatial frequency of fringes (by the same carrier frequency $f_{c}$) for all wavelengths of light and for all points of the extended source LS in OP.^2^ In principle, the system provides off-axis holography by the interference of the zeroth and first diffraction orders. Achromaticity and spatial invariance of the interference pattern follow from the fact that the diffraction grating is imaged onto output plane OP by ${\mathrm{OL}}_{2}$.^14^

The microscope is capable of imaging at various depths in a sample. Two approaches are available depending on the coherence of illumination.^15^^,^^16^ Full numerical refocusing is possible with highly coherent illumination, while with partially coherent illumination, the axial range of the refocusing gets reduced.^15^^,^^16^ On the other hand, low spatial coherence leads to the formation of an optical section in a broader sense,^15^ the thickness of which corresponds to the depth of field of the objective.^17^ Then, refocusing outside the section is only possible optically. This imaging mode is suitable for observation of adhering cells on a coverslip.

**Fig. 2:**
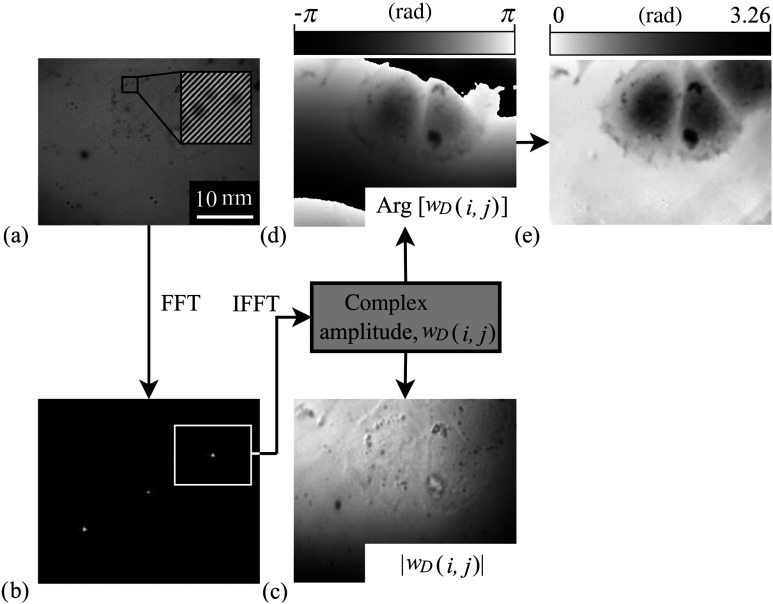
Image processing: (a) hologram of live cell, (b) the spatial-frequency spectrum with the windowing operation around the carrier frequency $f_{c}$, (c) amplitude image, (d) phase image, and (e) quantitative phase image.

The hologram captured by the CCD is processed by the computer in the following way, see Fig. 2. First, the Fourier transform of the hologram is computed. Then, the spatial-frequency spectrum of the object image is obtained by the windowing operation on the sideband of the spatial-frequency spectrum of the hologram (around the carrier frequency $f_{c}$). The zeroth spatial frequency is shifted to the center of the window and the spectrum is multiplied by the Hanning weight function. Finally, the image complex amplitude $w_{D}(i,j)$ is computed by the inverse Fourier transform, which provides the image phase $\mathrm{Arg}[w_{D}(i,j)]$ and the image amplitude $\left|w_{D}(i,j)\right|$. A quantitative phase image is obtained after applying an unwrapping procedure and background compensation.^18^^,^^19^

### 2.2 Theory

Spatially incoherent illumination requires correct lateral alignment of the images in both arms of the microscope. Temporally incoherent light demands adjusting the arms to the same length. In order to prove that the modulus of the reconstructed holographic signal is of significant value for the microscope alignment, we had to describe its dependence on the optical path length difference, ΔL, of the arms and on the mutual displacement of the images in the output plane OP that is described by the corresponding displacement vector $q_{f}=(x_{f},y_{f})$ in the object plane. The theoretical reconstructed holographic signal can be described by the following formula [see Eq. (3.20) in Ref. 15 for zero defocus]:

$$w(q_{t})=\iint_{-\infty }^{\infty }T_{t}(Q_{t})H_{t}(Q_{t})\exp(2\pi iQ_{t}\cdot q_{t})d^{2}Q_{t},$$ (1)

where $T_{t}(Q_{t})$ is the Fourier transform of the object transmission function [see Eq. (3.19) in Ref. 15], $H_{t}(Q_{t})$ is a two-dimensional (2-D) coherent transfer function of CCHM [see Eq. (3.21) in Ref. 15], $Q_{t}=(X,Y)$ is the transverse part of scattering vector, and $q_{t}=(x,y)$ is the Cartesian coordinate vector in the output plane divided by the microscope magnification. For sample-free object space, the relation $T_{t}(Q_{t})=\delta (X)\delta (Y)$ can be applied. Assuming a broad monochromatic light source and waves that are propagated at small angles to the optical axis, the modified equation can be obtained from Eq. (1) [see Eq. (6.1) in Ref. 15]. In our calculations, we retain the negative second power of the wave number K for its subsequent extension to the broadband source, so that

$$w(q_{t})=K^{-2}H_{t}(0,0)=K^{-2}\iint_{-\infty }^{\infty }S_{t}^{*}(K_{t};K)P_{t}(K_{t};K)d^{2}K_{t},$$ (2)

where $P_{t}(K_{t};K)=\mathrm{circ}[(K_{t}n)/(K\mathrm{NA})]$ is the 2-D pupil function of the objective lens $O_{1}$ with numerical aperture NA, circ(r) is a rotationally symmetrical function^20^ with the support of the radius 1, $S_{t}(K_{t};K)=i_{t}(K_{t})\mathrm{circ}[(K_{t}n)/(K{\mathrm{NA}}_{s})]$ is the 2-D effective pupil function of the illumination, where ${\mathrm{NA}}_{s}$ is the lowest of the numerical apertures of the condenser lenses and the objective lens $O_{2}$ in the reference arm, the function $i_{t}(K_{t})$ describes the distribution of the light intensity in the plane of the light source LS for the Köhler illumination,^15^ $K_{t}$ is the transverse part of K, $K_{t}=|K_{t}|$, and |K|=K=1/λ, λ is the wavelength and n is the refraction index in the object space of the objective lenses $O_{1}$ and $O_{2}$. Because ${\mathrm{NA}}_{s}\leq \mathrm{NA}$, the function $P_{t}(K_{t};K)$ can be removed from the integrand in Eq. (2). If the image fields of both arms are mutually shifted by the nonzero displacement vector $q_{f}=(x_{f},y_{f})$, the function $\exp(-2\pi iK_{t}\cdot q_{f})$ must be added to Eq. (2) [see Eq. (2.25) in Ref. 15]. Then,

$$w(q_{t};q_{f})=K^{-2}\iint_{-\infty }^{\infty }i_{t}(K_{t})\mathrm{circ}(\frac{K_{t}n}{K{\mathrm{NA}}_{s}})\times \exp(-2\pi iK_{t}\cdot q_{f})d^{2}K_{t}.$$ (3)

If we assume rotational symmetry of the intensity distribution $i_{t}(K_{t})$ of the light source LS, the Fourier transform [Eq. (3)] can be converted into the Hankel transform (see Ref. 15)

$$w(q_{t};q_{f})=2\pi \int_{0}^{{\mathrm{NA}}_{s}/n}i_{T}(\kappa )J_{0}(2\pi K\kappa \cdot q_{f})\kappa d\kappa ,$$ (4)

where $i_{T}(\kappa )$ is a radial intensity distribution, $\kappa =K_{t}/K$, κ=|κ|.

Equation (4) describes the reconstructed holographic signal for a spatially incoherent source characterized by the intensity distribution $i_{T}(\kappa )$. To also take into account the limited temporal coherence, suppose that the spectral properties of the source are described by the function $i_{K}(K)$, and the difference of the optical length of the arms in the image space is ΔL. The phase shift of the beam inclined by ψ is then $\Delta \phi_{\psi }=2\pi K\Delta L\;\cos\;\psi$ for the refractive index n≈1. Assuming that the sine condition holds for the microscope objectives, we get $\sin\;\psi ={\mathrm{NA}}_{s}/M\leq \mathrm{NA}/M$ for the beam of the maximum inclination ψ in the image space, where M is the magnification between the object plane and the output plane OP. The maximum ratio NA/M for the objectives used in CCHM has been found for NIKON CFI S Fluor 20×/0.75, where ψ=0.038  rad. Supposing λ=650  nm and maximum ΔL=100  μm, the difference of the phase shifts for the central beam and the maximum-inclination beam is $\Delta \phi_{0}-\Delta \phi_{\psi }=2\pi K\Delta L(1-\cos\;\psi )=0.21\pi \;\mathrm{rad}$. Hence, we can approximate the phase shift by $\Delta \phi_{0}$ for any inclination of the beam. After completing Eq. (4) by the spectral function $i_{K}(K)$ of the source and by the complex exponential depending on $\Delta \phi_{0}$ and by the integration over K, we get

$$w(q_{t};q_{f},\Delta L)=2\pi \int_{0}^{\infty }i_{K}(K)\times \exp(-2\pi iK\Delta L)\times \int_{0}^{{\mathrm{NA}}_{s}}i_{T}(\kappa )J_{0}(2\pi K\kappa \cdot q_{f})\kappa d\kappa dK.$$ (5)

Obviously, modulus $\overset{¯}{w}=|w|$ of the theoretical reconstructed holographic signal reaches its maximum for ΔL=0  μm and for $|q_{f}|=0\;\mu m$. For this reason, the modulus $\overset{¯}{w}$ emerged as a significant enough variable for the BReTA method of CCHM alignment.

### 2.3 Experiment

For verification of the BReTA methodology, the modulus $\overset{¯}{w}$ of the theoretically reconstructed holographic signal described by Eq. (5) was compared with experimental data. No specimen was inserted on the object plane. The measurement was performed with a spatially broad and spectrally narrowband light source. The source spectral function $i_{K}(K)$ was given by the manufacturer data (Thorlabs) of the interference filter FB650-10 (λ=650  nm, FWHM=10  nm); its radial intensity distribution $i_{T}(\kappa )$ was approximated by a Gaussian distribution^21^ with the proportional reciprocal standard deviation α=2.5 that was found by fitting the theoretical curve to the experimental data. To eliminate the noise, the modulus of the measured reconstructed holographic signal was averaged over the whole image field as follows:

$${\overset{¯}{w}}_{D}=\overset{N_{I}-1}{\underset{i=0}{\sum }}\overset{N_{J}-1}{\underset{j=0}{\sum }}\frac{\left|w_{D}(i,j)\right|}{N_{I}N_{J}},$$ (6)

where $N_{I}$ and $N_{J}$ are sizes of $w_{D}(i,j)$.

**Fig. 3:**
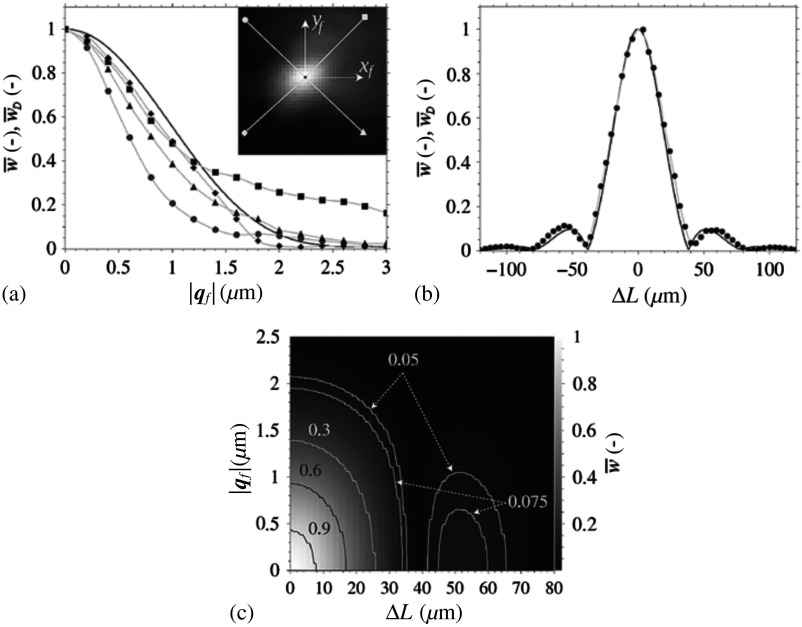
Comparison between measured values of moduli of theoretically reconstructed holographic signal $\overset{¯}{w}$ (solid line) and measured reconstructed holographic signal ${\overset{¯}{w}}_{D}$ (symbol lines). (a) Dependence on $\left|q_{f}\right|$ for identically long arms of the interferometer ΔL=0  μm. Profiles from the $q_{f}=(0,0)\;\mu m$ to four directions are shown by the symbol lines. (b) Dependence on ΔL for $|q_{f}|=0\;\mu m$. (c) Contour graph of the dependence of theoretically reconstructed holographic signal modulus $\overset{¯}{w}$ on $\left|q_{f}\right|$ and ΔL. The values of $\overset{¯}{w}$ and ${\overset{¯}{w}}_{D}$ are normalized.

Figure 3(a) shows the comparison of dependences of reconstructed holographic signal moduli $\overset{¯}{w}$ and ${\overset{¯}{w}}_{D}$ on $\left|q_{f}\right|$. Measured curves slightly differ mutually and from the theoretical curve. It is due to the optical aberrations of the output lens ${\mathrm{OL}}_{2}$, which is located behind the DG in the reference arm. Light diffracted to the first order passes through the border of aperture ${\mathrm{OL}}_{2}$ (in comparison to the axial propagation in the object arm). Moreover, shifting the objective lens $O_{2}$ laterally leads to transversal displacement of the diffracted beam in the aperture ${\mathrm{OL}}_{2}$. In contrast, the measured values of modulus of the reconstructed holographic signal on Fig. 3(b) perfectly fit the theoretical values. Measurement was performed for the same light source and for $|q_{f}|=0\;\mu m$ by changing ΔL.

**Fig. 4:**
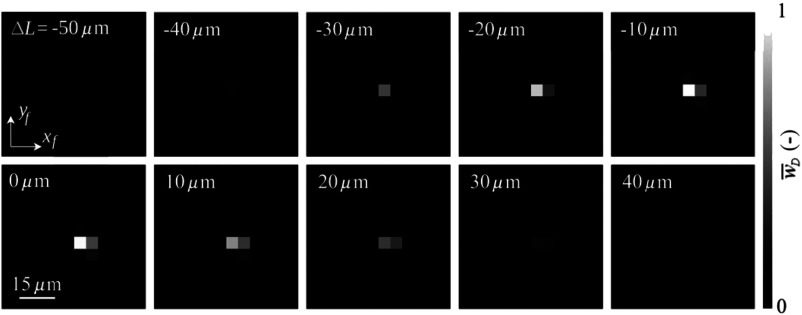
Measured dependence of the modulus ${\overset{¯}{w}}_{D}$ of reconstructed holographic signal on the displacement vector $q_{f}=(x_{f},y_{f})$ and on the optical path difference ΔL. Gray levels in the pixels (small squares) of each subimage represent ${\overset{¯}{w}}_{D}$ average on the field of view for various $q_{f}$ and constant ΔL indicated in the subimage. Average values of ${\overset{¯}{w}}_{D}$ are normalized by its maximum over the complete set of measurements.

The theoretical reconstructed holographic signal modulus $\overset{¯}{w}$ independence between $\left|q_{f}\right|$ and ΔL is shown in Fig. 3(c). Because of its symmetry, it is displayed only in the first quadrant. Its global maximum ${\overset{¯}{w}}_{\max}$ is apparent in the origin. Local maximum ${\overset{¯}{w}}_{l,i}$ along the axis ΔL is the consequence of the form of the source spectral function $i_{K}(K)$. Behavior in the direction of the axis $\left|q_{f}\right|$ is smooth with no side lobes as a result of the Gaussian form of the intensity $i_{T}(\kappa )$. The modulus $\overset{¯}{w}$ has the maximum values on the axis ΔL for constant $\left|q_{f}\right|$ and on the axis $\left|q_{f}\right|$ for constant ΔL, see also experimental data in Fig. 4.

**Fig. 5:**
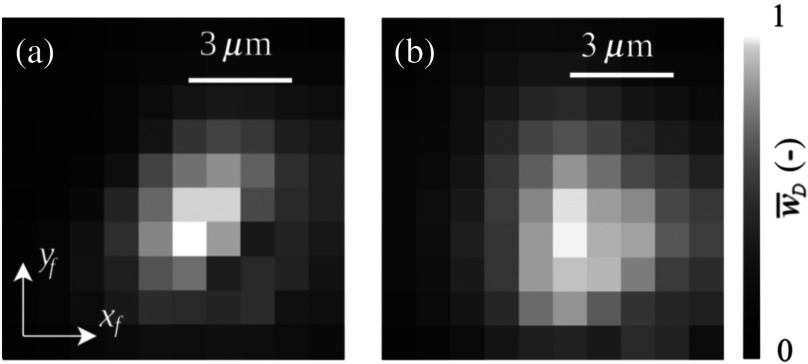
Measured dependence of the modulus ${\overset{¯}{w}}_{D}$ of reconstructed holographic signal on the displacement vector $q_{f}=(x_{f},y_{f})$ (${\overset{¯}{w}}_{D}$ measured and represented as in Fig. 4, ΔL=0  μm) for (a) spectrally narrowband light source (λ=650  nm, FWHM=10  nm) and (b) broadband light source. The values of ${\overset{¯}{w}}_{D}$ are normalized; the normalized maximum value ${\overset{¯}{w}}_{D}<1$ for ΔL≠0  μm.

To demonstrate that the measured reconstructed holographic signal modulus, ${\overset{¯}{w}}_{D}$, has a strong maximum at $|q_{f}|=0\;\mu m$ and ΔL=0  μm even with broadband light source, a measurement was carried out similar to the previous case, but with the narrowband filter removed. Figure 5 compares measurements containing the maximum value ${\overset{¯}{w}}_{D,\max}$ for the case of spectrally narrowband and broadband light source. Broadband illumination leads to a broader peak around ${\overset{¯}{w}}_{D,\max}$ [Fig. 5(b)] in comparison with spectrally narrowband illumination [Fig. 5(a)]. Extension of the peak is caused by the superposition of peaks related to different wavelengths, which do not overlap ideally due to chromatic aberration of the optical imaging system. Therefore, the measured reconstructed holographic signal modulus ${\overset{¯}{w}}_{D}$ is a significant enough value for BReTA method in the case of spectrally narrowband light, as well as broadband light.

### 2.4 Basic Realignment Three-Dimensional Algorithm Method

#### 2.4.1 Setup adjustment to basic realignment three-dimensional algorithm method

**Fig. 6:**
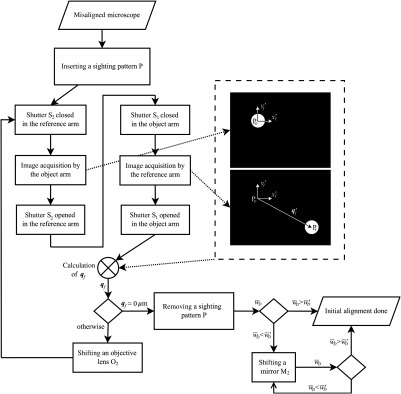
The flow chart of the procedure of initial alignment.

Several essential elements of the microscope setup had to be motorized (see Fig. 1) for experimental implementation of the BReTA method. The XY linear stage of the objective $O_{2}$ was motorized to vary $q_{f}$; the holder of the mirror $M_{2}$ was mounted on a linear stage for longitudinal movement ΔL. A sighting pattern was placed in the plane conjugated with the image plane, and computer-controlled shutters $S_{1}$ and $S_{2}$ were placed in the reference and object arms. The algorithm of the BReTA method described in this part is based on the measurement of the reconstructed holographic signal modulus ${\overset{¯}{w}}_{D}$ (hereafter the signal ${\overset{¯}{w}}_{D}$), see Fig. 6.

#### 2.4.2 Initial alignment

This procedure is used for correction of large misalignment typical for the microscope startup. First, the proper shifts of the mirror $M_{2}$ and objective lens $O_{2}$ are found with the aim to approach the area of the global maximum of ${\overset{¯}{w}}_{D}$. The final value of the signal must be higher than the threshold value ${\overset{¯}{w}}_{D}^{\prime }=k{\overset{¯}{w}}_{D,l_{1}}$, where ${\overset{¯}{w}}_{D,l_{1}}$ is the value of the first side maximum and k is the safety factor. In the beginning of the process, the sighting pattern P is inserted into the field plane and imaged to the object plane (the plane of the specimen SP and of the reference object RO) and finally to the output plane OP. The centers of images are mutually shifted by

$$q_{f}^{\prime }=(x_{P_{r}}^{\prime }-x_{P_{o}}^{\prime },y_{P_{r}}^{\prime }-y_{P_{o}}^{\prime }).$$ (7)

The coordinates of the centers of the object and reference arm images are found by closing the shutters $S_{1}$ and $S_{2}$, respectively, and measuring the position of the sighting pattern centers $P_{o}=[x_{P_{o}}^{\prime },y_{P_{o}}^{\prime }]$, $P_{r}=[x_{P_{r}}^{\prime },y_{P_{r}}^{\prime }]$, respectively. The required shift $q_{f}$ is then expressed as follows:

$$q_{f}=\frac{q_{f}^{\prime }}{M}=\frac{\left(x_{P_{r}}^{\prime }-x_{P_{o}}^{\prime },y_{P_{r}}^{\prime }-y_{P_{o}}^{\prime }\right)}{M},$$ (8)

where M is the magnification between the object plane and the output plane OP. Subsequently, the objective lens $O_{2}$ is shifted by $-q_{f}$. This sequence is repeated until $|q_{f}|\approx 0\;\mu m$. Then, the elements P, $S_{1}$, and $S_{2}$ are removed.

After this process, the value of the signal ${\bar{w}}_{D}$ is tested. If ${\bar{w}}_{D}$ is greater than ${\overset{¯}{w}}_{D}^{\prime }$, the process ends. If not, ΔL is changed in the direction of increasing signal value ${\bar{w}}_{D}$ by moving the mirror $M_{2}$, until the condition is met.

#### 2.4.3 Advanced alignment procedure

In this procedure, the elements $O_{2}$ and $M_{2}$ are moved sequentially to search for the maximum value of signal ${\overset{¯}{w}}_{D,\max}$. Because the area of the global maximum is smooth, it is possible to choose any sequence of independent processes for search of the value ${\overset{¯}{w}}_{D,\max}$, which are described in this section.

**Fig. 7:**
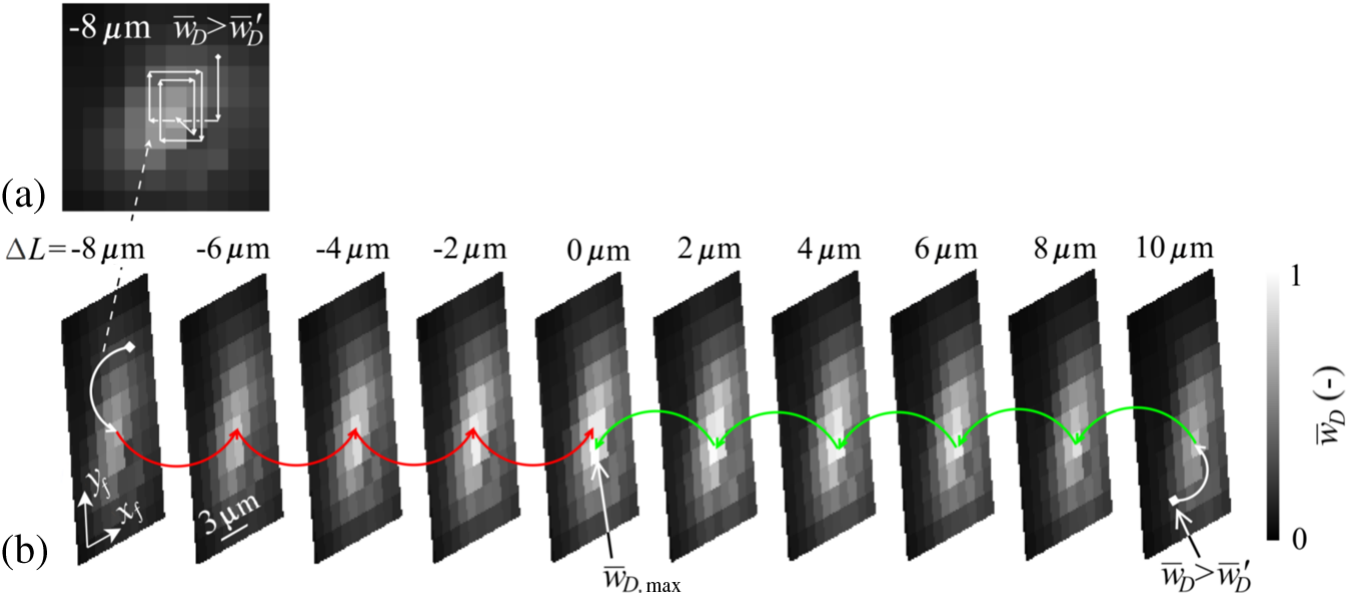
Illustration of the advanced alignment procedure. The modulus ${\overset{¯}{w}}_{D}$ of holographic signal is measured, normalized, and represented as in Fig. 4. (a) First, the heuristic algorithm searches for the local maximum value of ${\bar{w}}_{D}$ changing $q_{f}$ and keeping ΔL constant. This is done by lateral movements of the microscope objective $O_{2}$. (b) Then, the global maximum value ${\overset{¯}{w}}_{D,\max}$ is found changing ΔL by the axial movement of the mirror $M_{2}$ in the direction of increasing values of ${\overset{¯}{w}}_{D}$. The values of the reconstructed holographic signal modulus (i.e., values of the signal) ${\overset{¯}{w}}_{D}$ are normalized.

The first process deals with searching the greatest value of signal ${\bar{w}}_{D}$ by changing the microscope objective $O_{2}$ position while maintaining ΔL constant. It can be performed in many ways. For example, by a 2-D scanning of objective lens $O_{2}$ around its current position; the resulting scans can be seen in Fig. 7(b). This is a very easy and robust way. Another faster possibility is to use a heuristic algorithm with appropriate termination condition. Algorithm of this process is illustrated in Fig. 7(a). It determines the value of signal ${\overset{¯}{w}}_{D,1}$ in the initial position of $O_{2}$. Its position $q_{f}$ is then changed by a small defined step in any direction, and the obtained value of signal ${\overset{¯}{w}}_{D,2}$ is compared with ${\overset{¯}{w}}_{D,1}$. If ${\overset{¯}{w}}_{D,2}<{\overset{¯}{w}}_{D,1}$ is true, algorithm executes the next step right toward the original step direction; otherwise, the step is performed in the original direction. If the exit condition is not true, the value of signal ${\overset{¯}{w}}_{D,3}$ is obtained and again compared with the previous value of the signal ${\overset{¯}{w}}_{D,2}$. The termination condition can be selected according to the situation, whichever occurs first. Optionally, the combination of the conditions may be required. The termination can be based on multiple detection of the greatest signal ${\overset{¯}{w}}_{D}$ in the same position, when the algorithm is repeatedly going through the same coordinates, or on the calculation of the variational coefficient from the last several values of signal ${\overset{¯}{w}}_{D}$. The variation coefficient must be close to one.

The second process finds the value of the signal ${\overset{¯}{w}}_{D,\max}$ by moving the mirror $M_{2}$ in the direction of the increased values of the signal ${\overset{¯}{w}}_{D}$ [see Fig. 7(b)]. It seeks such a position of mirror $M_{2}$ that corresponds to ΔL=0  μm.

Both processes together lead to the alignment defined by ΔL=0  μm and $|q_{f}|=0\;\mu m$.

#### 2.4.4 Testing of both procedures

For testing the procedures, Nikon objective lenses 10×/0.30 were used. Broadband light from the source was filtered by the interference filter (λ=650  nm, FWHM=10  nm). Holograms were captured by camera XIMEA MR4021MC-BH. The choice of the minimum alignment steps in the lateral ($q_{f}$) and axial (ΔL) directions depends on the targeted signal level, according to the graph in Fig. 3(c). As it will be shown in Sec. 2.4.5, the sufficient signal level resulting in a good-quality QPI is ∼90%. The corresponding acceptable misalignment is 0.5  μm in $q_{f}$ and 10  μm in ΔL [see Fig. 3(c)]. Hence, for the initial alignment, we chose the step 10  μm of ΔL, while $q_{f}$ is set approximately to zero by overlapping the sighting pattern images formed in reference and object arm. The robust scanning method runs laterally within 5×5 positions with the steps 0.5  μm of $x_{f}$ and $y_{f}$, while the axial step of ΔL could be fined down to 1  μm. As the movement of the mirror $M_{2}$ is linear and unidirectional, this correction did not noticeably prolong the axial scanning, in contrast to the situation in lateral directions.

**Fig. 8:**
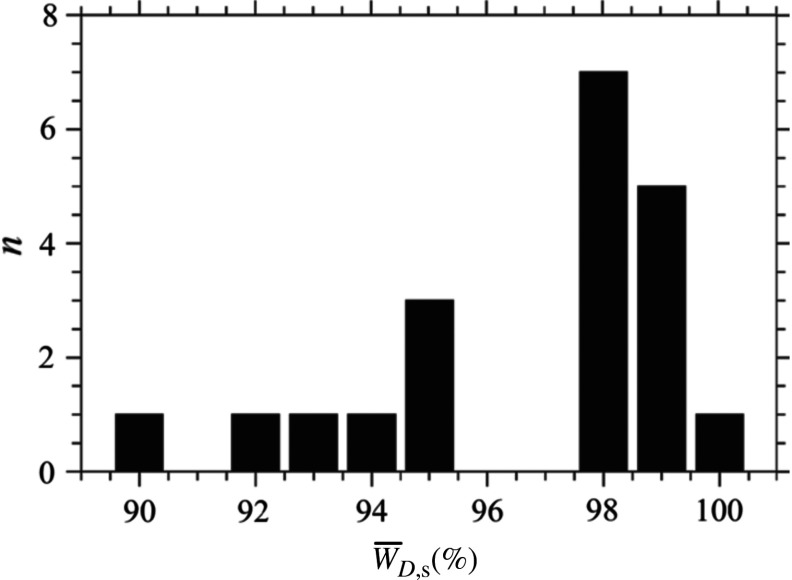
Histogram demonstrating success rate of the tested procedures.

The testing method consists of repeated misalignment of the microscope and subsequent activation of both alignment procedures. The microscope was randomly misaligned by the shift of the objective lens $O_{2}$ and by moving the mirror $M_{2}$ by a distance 100  μm. Twenty independent measurements were performed. The average time required to approach the aligned state of the microscope was 136 s. Figure 8 shows the results of automated alignment in comparison with the manually obtained (i.e., the most precise) result. The relative values of signal ${\overset{¯}{w}}_{D,s}=({\overset{¯}{w}}_{D,\max}/{\overset{¯}{w}}_{D,\max,m})\cdot 100\%$, where ${\overset{¯}{w}}_{D,\max,m}$ is the value of the signal obtained by manual alignment, are on the x axis. The y axis is the count axis.

It is obvious that tested procedures of the BReTA method always resulted without fail with a high success rate.

#### 2.4.5 Alignment procedure for a long-term experiment

**Fig. 9:**
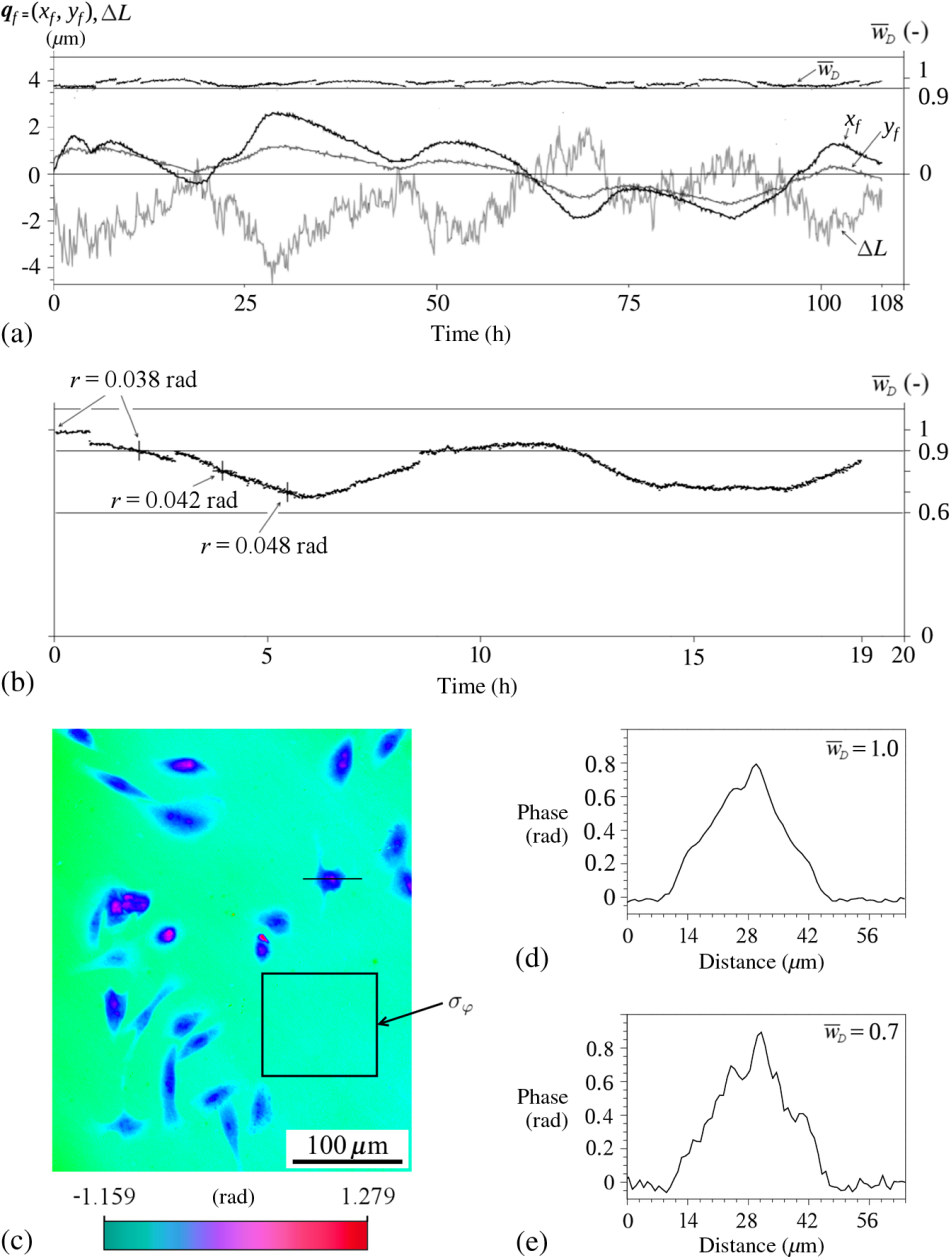
(a) Long-term maintenance of high values of the measured holographic signal ${\overset{¯}{w}}_{D}$ and changes of $q_{f}=(x_{f},y_{f})$ and ΔL, which show the corresponding shifts of the objective lens $O_{2}$ and of the mirror $M_{2}$, respectively. The values of the signal ${\overset{¯}{w}}_{D}$ are normalized. (b) The time dependence of the signal ${\overset{¯}{w}}_{D}$ without the long-term maintenance procedure. Measured phase resolution r in selected points of the graph is indicated. (c) Quantitative phase image of rat sarcoma cells obtained with 90% value of the holographic signal [r=0.038  rad, see the graph (b)]. The measurement of $\sigma_{\varphi }$ was performed within the window (22,000 pixels) plotted on the image. (d) Phase distribution along the line depicted in (c) for 100% value of the holographic signal with the long-term maintenance procedure activated. (e) Phase distribution along the same line with the apparent noise increase after the procedure deactivation and the holographic signal drop to 70% value.

During the long-term experiments, the value of signal ${\overset{¯}{w}}_{D}$ is influenced by temperature changes or vibrations. Therefore, we extended the BReTA method for long-term maintenance of the maximum value of the signal ${\overset{¯}{w}}_{D}$. The procedure periodically activates both the transverse stages for the movement of the microscope lens $O_{2}$ and the axial stage to move the mirror $M_{2}$. They are moved symmetrically around the current positions of active elements $O_{2}$ and $M_{2}$ by given steps. The procedure compares the original value of signal ${\overset{¯}{w}}_{D,\max}$ and values ${\overset{¯}{w}}_{D}$ in adjacent positions of an active element. The active element is always moved to the position with the maximum value of signal ${\overset{¯}{w}}_{D}$. In Fig. 9(a), the time dependence of the signal values ${\overset{¯}{w}}_{D}$ during the run of the procedure is shown. The procedure changes the positions of the microscope objective $O_{2}$ with the step 100 nm of $x_{f}$ and $y_{f}$ and the optical path difference ΔL of arms (the position of the mirror $M_{2}$) with the step 200 nm. As the procedure is running during observations, the minimum repeatable steps of the linear stages have been chosen to avoid visible changes of the image recorded. Figure 9(b) shows the time dependence of the signal ${\overset{¯}{w}}_{D}$ without the alignment procedure running. The signal is inconsistent and too low most of the time. The decrease of the signal makes the phase noise higher, thereby impairing the QPI resolution. We define the QPI resolution as $r=2\sigma_{\varphi }$, where $\sigma_{\varphi }$ is the standard deviation of the background phase values measured in the window drawn in Fig. 9(c). The decrease of the signal to 90% has no measurable effect on the resolution; for its lower values, the resolution is elevated measurably [see Fig. 9(b)]. The increase of the noise in the phase image of real object is demonstrated in Figs. 9(d) and 9(e).

The microscope was subjected to temperature changes of environment with fluctuations of tenths of centigrade. It is obvious that the procedure successfully maintained the aligned state of the microscope.

## 3 Conclusions

We have developed a unique set of procedures constituting the original BReTA. The BReTA method allows for automated alignment of CCHM based on maximizing the value of modulus $\overset{¯}{w}$ of the measured holographic signal. For exerting control over this parameter, some alignment elements of the original CCHM setup had to be motorized. The method consists in the initial alignment of the microscope in order to find the required minimal interference signal. The holographic signal is then optimized by searching the best alignment corresponding to the maximum signal. This maximum can be subsequently maintained by small changes in the alignment during a long-term experiment. All procedures were programmed in LabView and C++, and they are being used in the multimodal holographic microscopes produced by TESCAN ORSAY HOLDING a.s. The automatic BReTA method offers easy alignment of the microscope for ordinary users. However, it is essential for managing long-term QPI observations/measurements of live cells activity, which is the primary assignment of holographic microscopy. The BReTA method described in this article is patent pending.^22^
